# Aggressive Mimicry and the Evolution of the Human Cognitive Niche

**DOI:** 10.1007/s12110-023-09458-y

**Published:** 2023-09-06

**Authors:** Cody Moser, William Buckner, Melina Sarian, Jeffrey Winking

**Affiliations:** 1 Department of Cognitive and Information Sciences, University of California, Merced, Merced, CA USA; 2 Human Systems and Behavior Lab, Department of Anthropology, Boston University, Boston, MA USA; 3 Department of Anthropology, University of California, Davis, Davis, CA USA; 4 Department of Anthropology, Texas A&M University, College Station, TX USA

**Keywords:** Mimicry, Deception, Cognitive evolution, Language evolution, Hunting, HRAF

## Abstract

The evolutionary origins of deception and its functional role in our species is a major focus of research in the science of human origins. Several hypotheses have been proposed for its evolution, often packaged under either the Social Brain Hypothesis, which emphasizes the role that the evolution of our social systems may have played in scaffolding our cognitive traits, and the Foraging Brain Hypothesis, which emphasizes how changes in the human dietary niche were met with subsequent changes in cognition to facilitate foraging of difficult-to-acquire foods. Despite substantive overlap, these hypotheses are often presented as competing schools of thought, and there have been few explicitly proposed theoretical links unifying the two. Utilizing cross-cultural data gathered from the Human Relations Area Files (HRAF), we identify numerous (*n* = 357) examples of the application of deception toward prey across 145 cultures. By comparing similar behaviors in nonhuman animals that utilize a hunting strategy known as aggressive mimicry, we suggest a potential pathway through which the evolution of deception may have taken place. Rather than deception evolving as a tactic for deceiving conspecifics, we suggest social applications of deception in humans could have evolved from an original context of directing these behaviors toward prey. We discuss this framework with regard to the evolution of other mental traits, including language, Theory of Mind, and empathy.

**Supplementary Information:**

The online version contains supplementary material available at 10.1007/s12110-023-09458-y.

Deception is rarer in nature than honest communication (Dawkins & Krebs, 1978). There are benefits to deceiving others, and, conversely, to being able to detect others’ deception (Wallace, 1973). Although the basis for communication lies in the use of signals by one organism to manipulate the behavior of others, counterbalancing selective forces ensure that most communication within a species is honest. Despite its rarity, the extensive use of deception has been noted as a hallmark of human cognition. In addition to the game theoretical question of how deception can arise and be maintained, another question continues to puzzle evolutionary scientists: Why is it that humans, in particular, lie so well?

Many proposals argue that the fitness functions of some of our cognitive abilities—such as our ability to lie—are to manipulate and outmaneuver conspecifics, forming a Machiavellian Intelligence (Bereczkei, 2018; Lucas et al., 2018; Whiten & Byrne, 1988a). “Machiavellian Intelligence” refers to individuals’ abilities to socially outmaneuver others to achieve a preferred outcome. Packaged as part of the Social Brain Hypothesis, a popular view is that many of our cognitive features, including our large brain sizes and our use of deception, evolved to accommodate the cognitive demands associated with managing social relationships within our large social groups (Barton & Dunbar, 1997; Dunbar, 1998). In this account, increases in group sizes led to the selection for novel cognitive faculties that improved relative abilities to socially compete and cooperate within these increasingly complex social systems. This hypothesis therefore argues that much of our cognition has been shaped by co-evolution with our conspecifics, which may have included the ability to lie (Byrne & Whiten, 1992).

Another popular hypothesis for the evolution of human cognitive traits is the foraging brain hypothesis proposed by Kaplan et al. (2000). Kaplan et al. posit that human cognitive prowess arose as a result of large-scale climatic shifts during the Pleistocene in which early humans shifted from a relatively easy, chimpanzee-like foraging niche to a more cognitively demanding one. In this scenario, the expansion of the human brain is seen as the result of ecological rather than endogenous factors, such as intraspecific competition or runaway sexual selection. Instead, our behaviors became more flexible to solve more challenging and variable problems in our environment with higher payoffs. Behaviors which may have arisen during this time include better memory and problem-solving, a greater reliance on social information from our conspecifics, and the emergence of food sharing and cooperation. Although neither hypothesis should be taken as exclusive of the other, they differ in their points of emphasis and their relevance for the evolution of deception. Through the social brain hypothesis, we have an account which largely considers endogenous social factors as relevant to the evolution of human cognitive traits, and through the foraging brain hypothesis, we have an account which is largely agnostic about deception altogether.

Deception in animals takes the form of at least two types: tactical deception and aggressive mimicry. In cases of tactical deception, animals either withhold or falsify information using otherwise “honest” signals from their standard repertoire to deceive other individuals (Whiten & Byrne, 1988b). Most, if not all, cases of intraspecific deception take this form. Many examples from nonhuman primates include the use of alarm calls to distract conspecifics from resources, to distract aggressors, and to hide resources or mates from other individuals (Cheney & Seyfarth, 1990): squirrels deceptively create false caches of food in the presence of conspecifics to prevent detection of real caches (Steele et al., 2008), and corvids have been found to track the eyesight of other corvids to hide food behind barriers (with this behavior extending even to solitary nutcrackers) (Bugnyar & Heinrich, 2005; Clary and Kelly, 2011; De Kort and Clayton, 2005; Emery and Clayton, 2004). Separately, in aggressive mimicry, predators “communicate with their prey by making signals to indirectly manipulate prey behavior” (Jackson & Cross, 2013:161). Common examples in nature include angler fish evolving appendages to entrap their prey, predatory species of firefly mimicking the lighting patterns of other species as lures, and carnivorous pitcher plants which attract insects utilizing nectar (Nelson, 2014).

Although the lens of scientific inquiry has long been turned on the use of *tactical* deception by humans, few systematic attempts have been made to categorize the forms and prevalence of aggressive mimicry in human contexts. As such, this study attempts to index, categorize, and record the incidents of human aggressive mimicry in both large- and small-scale societies using the Human Relations Area Files database, composed of ethnographic texts from 361 societies on every human-populated continent, with the addition of Oceania as a geographic region.

## Cognitive Aggressive Mimicry in Animals

In order to make a direct comparison to humans, we focus on nonanatomical examples of aggressive mimicry, in a form of aggressive mimicry referred to as *cognitive* aggressive mimicry (Jackson & Cross, 2013). While anatomical aggressive mimicry has evolved in numerous organisms, including fish, snakes, and mammals, a cognitive form comprising only mimicked *behavior* is much rarer, being consistently documented in only a few species.

Among habitual mimicry in birds, two cases have been consistently observed and a third case comes from historical reporting. In the first case, field reports from multiple species of heron (family *Ardeidae*) and egrets (*Egretta garzetta*) show that these species employ an active form of bait-fishing in order to catch aquatic prey (Post et al., 2009). Displaying some form of delayed gratification, these birds are known to use a lure such as bread, feathers, or insects to attract more desirable prey to the edge of the water. The second case comes from the relatively asocial shrikes (family *Laniidae*), which have been reported as far back as medieval times to vocally mimic their prey (Atkinson, 1997). Writing about shrikes in 1575, the English poet Turberville noted,

She will stand at perch upon some tree or poste, and there make an exceedingly lamentable crye. . . . All to make other fowles to thinke that she is very much distressed. . . whereupon the credulous sellie birds do flocke together at her call. If any happen to approach near her, she. . . ceazeth on them, and devoureth them (ungrateful subtill fowle). (Turberville, 1575:73)

Field experiments by Aktinson (1997) indicate that northern shrikes (*Lainus excubitor*) in Canada use acoustic mimicry to imitate the alarm calls of smaller birds in winter to lure them toward hidden perches. Although they are asocial, shrikes share a recent common ancestor with the *Corvidae*, a family long characterized for its intelligence (Jønsson et al., 2016). Two species of corvid, blue jays (*Cyanocitta cristata*) and Steller’s jays (*Cyanocitta stelleri*), have been noted to employ a defensive form of mimicry while mimicking the calls of their own avian predators (Hailman, 2009; Tippin, 2017).

Finally, in the most phylogenetically unique example of mimicry, spiders of the genus *Portia* utilize deception to acquire and consume much larger, more venomous prey, especially wolf spiders (Jackson & Blest, 1982). Deception in these instances often takes the form of faking vibrational signals on the webs of prey, taking advantage of the wolf spiders’ mechanism used to detect the presence of their own prey, potential mates, and rivals. Such cases have been documented in a wide array of other spiders, including several jumping spider genera (*Brettus, Cyrba*, and *Gelotia*) and in the pirate spiders of the family *Mimetidae* (Jackson, 1992). Similar examples have also been described in spider-eating assassin bugs (Wignall & Taylor, 2011).

In addition to these habitual examples, other incidental examples of cognitive aggressive mimicry have been described. For example, multiple Central and South American big cats (*Puma concolor, Panthera onca*, and *Leopardus pardalis*) have been reported by Amazonian inhabitants to mimic the vocalizations of pied tamarins (*Saguinus bicolor*), a claim further supported by field observations (de Oliveira Calleia et al., 2009). Two crocodilian species (*Crocodylus palustris* and *Alligator mississippiensis*) have also been observed to use twigs and sticks for camouflage and as hunting lures, primarily during the nest-building season of their prey (Dinets et al., 2015).

## Cognitive Aggressive Mimicry in Humans

The use of deception in hunting has been documented as a common practice by contemporary hunters. As rare as aggressive mimicry is in the animal kingdom, anecdotally it appears to be commonly employed by humans. Hunters in the Southeastern United States regularly utilize “hawk whistles” while hunting rabbits and squirrels by using a high-pitched whistle to mimic the sound of a hawk to freeze their prey in place (Angier, 2016). Archaeological evidence of the use of decoys in hunting contexts dates back in the Western Hemisphere at least 2,000 years (Hitchcock et al., 2019) and in Micronesia for at least 3,000 years (Carson & Hung, 2021). The explicit copying of avian vocalizations by human hunters and speakers even has its own name: warblish (Sarvasy, 2016:766), defined as “The phenomenon of vocal imitation of avian vocalizations by humans, using existing non-onomatopoeic word(s), as with English *who cooks for you?* (for the barred owl call) and *Chicago!* (for the California quail call); or a particular vocal imitation using existing word(s).” Given the anecdotal accounts of aggressive mimicry among humans, a natural question which follows is to what extent this behavior is practiced across cultures. This study therefore seeks to assess the cross-cultural facets of deception aimed at prey and the evolutionary implications of such forms of deception.

## Methods

We used the online Human Relations Area Files database to compare occurrences of aggressive mimicry across human societies with differing locations and subsistence strategies (Fischer & Ember, 2018; Naroll, 1967). The sample is composed of ethnographic texts from 326 societies (at the time of this search) on every human-populated continent, with the addition of Oceania as a geographic region. Societies are split by both regions within these continents and subsistence type (e.g., hunter-gatherer, pastoralist, industrial diaspora). As a large database composed of thousands of ethnographic texts, the Human Relations Area Files’ online component (eHRAF) lends itself to broad corpus research since querying specific terms will extract not only the term itself, but the entire document it is embedded within. Nevertheless, several caveats are worth noting. While this method can be used to establish, at a minimum, the presence of a specific trend, generally speaking the questions of researchers who use the database for broad searches, as in our case, are not the same questions the ethnographers had in mind (if they had any) when collecting their data. Whereas some ethnographers may have focused on hunting methods among a specific society and extensively recorded cases of deception against prey, others may have simply noted the practice in the course of other work. Thus, comparisons between societies about the scale of tactical deception should be avoided. In addition, an absence of evidence cannot be assumed to indicate a lack of these hunting methods.

We identified the use of aggressive mimicry in the Human Relations Area Files with the root of the following terms: deceive, deception, decoy, imitate, lure, and mimic. With eHRAF’s system for querying searches, we used the items deceiv*, deception, decoy*, imitat*, lure*, luring, and mimic*. Since we were primarily interested in the transmission of false signals as opposed to the withholding or masking of cues, as in the case of camouflage, we avoided terms directly related to camouflage, hunting blinds, and scent masking such as trap, bait, and snare (Bradbury & Vehrencamp, 1998; Lloyd, 1983). eHRAF additionally allows the filtering of search terms by subject. Subjects were limited to Agriculture, Animal Husbandry, Food Consumption, Food Quests, and Ideas about nature and people. To comprehensively assess the context of each category of deception, we collected data on types of prey captured/killed: fishing, large mammal (and type of mammal), small mammal, carnivore (and type of carnivore), birds, and primates. The social context of the lure was also ascertained based on the surrounding text: individual, group, or unknown.

In addition, the sensory system being exploited by humans in each context was documented: acoustic lures, baiting, fire fishing, olfactory, visual, a mix of any types, and ambiguous. These were determined based directly on the manner by which the lure was communicated to prey. Because many of the fishing samples consist of different forms of baiting where the sensory context may not be comparable with terrestrial modalities (i.e., to avoid the question of whether, from the perspective of the hunter, fish and birds or mammals perceive acoustic or baited signals the same “way”), we analyze the dataset both with and without fishing included.

Acoustic mimicry is typically accomplished vocally, although in some societies this is achieved with the use of an instrument, such as among the Assiniboine of North America, who use whistles made of wood to lure deer and elk (Denig & Hewit, 1930). These examples are not limited to terrestrial prey. One acoustic lure for fish among the South American Ticuna is described as follows: “With the ball he strikes the surface of the water, thus imitating the fall of fruits, in order to attract certain fish—especially tambaquís (*Colossoma bidens*) and pacú (*Myteles* sp.)” (Nimuendajú, 1952).

Visual mimicry commonly involves the use of special clothing, items, or movements to decoy the animal. For example, among the Chukchi of Siberia, a hunter wears a sealskin hat imitating a seal’s head, wields a special scraper with seal’s claws attached, and moves while imitating the movements of a seal, periodically scratching the ice with the claws, until he is close enough to strike with a harpoon throw (Antropova & Kuznetsova, 1964).

Vibrational cues are those which use natural vibrations in the ground, in trees, or otherwise in the environment to attract prey. An example of a vibrational cue comes from the Kimam Papuans, who used vibrations for kangaroos by “stamping on the ground from time to time while approaching their game, in imitation of a jumping kangaroo” (Serpenti, 1965).

Baiting cues were cues in which some form of bait was provided to animals, such as food or a potential competitor. For example, one form of baiting using food was described among the Iroquois, where there was “a close relationship between the old Seneca custom of sacrificing the first-killed deer to the meat-eating birds of prey and the widespread American Indian technique of luring down birds to shoot them or take them by pit trapping” (Fenton, 1953). Relatedly, mates or potential competitors may be used, as among the Eastern Toraja of Southeast Asia: “First the Toradja sees to it that he has tamed a female buffalo which is to help him with the catching; such a decoy animal is called *poanda*. People set out during the day and also often at night by moonlight. The buffalo cow is held on a line fastened to the nose ring” (Adriani & Kruijt, 1951).

Fishing cues generally took the form of line fishing, in which bait is placed on a hook or tied to a line and sent out into the water. Where the bait is explicitly mentioned, these examples are coded as “baiting,” but where it is unspecified, we code it as fishing. Since much of the dataset with regard to baiting is skewed by the presence of fishing examples, we analyze the results both with and without these examples. One special form of, presumably, visual mimicry is the use of fire to lure fish, which is also coded separately. As noted among the Micronesian Yapese people, “The period for catching flying fish, in May and June, represents a week-long festival, during which all the able-bodied men go out to sea night after night in whole flotillas of canoes, lure the schools from the water by torchlight, and snatch the dazzled sea-inhabitants by the thousands out of the air with long-handled hoop-nets, like butterflies” (Müller, 1917). This form of mimicry or visual baiting was coded separately as fire fishing.

A mixed form of exploitation involved any two or more of the other types of contexts, often in the form of decoys. For example, among the Asian Eastern Toraja, “Here he [imitates] the sounds of wood pigeons (lebago, togooe), in order to lure these birds. For this purpose he also has with him a decoy pigeon on a perch with a long pole” (Adriani & Kruijt, 1951). Textual descriptions for most of the examples in eHRAF can be found in the ESM.

Finally, the directedness of the lure, defined as luring the animal directly using a signal from the body, were also ascertained as direct (using the body), indirect (using another animal, as in the case of baiting, or a tool), or ambiguous. For example, one description of the Amazonian Sirionó was coded as both a direct and an indirect acoustic lure for alligators: “Newborn alligators are sometimes used by hunters to attract the mother. When a young alligator is caught it begins to cry for its mother, who, upon hearing it, comes running out of the water to retrieve it. The hunter, waiting on shore, strikes the mother over the head with a club as she comes up the bank. By imitating a young alligator, a hunter can often produce the same result” (Holmberg, 1950).

## Results

The results of the eHRAF term search can be found in the ESM and are summarized here. The six terms Deceiv*, Deception, Decoy*, Imitat*, Lure*/Luring, and Mimic* yielded 324, 111, 327, 1365, 603, and 131 paragraphs, respectively. Of these, Deceive* yielded 18 results of aggressive mimicry, Deception yielded 5, Decoy* yielded 109, Imitat* yielded 80, Lure*/Luring yielded 146, and Mimic* yielded 8, for a total of 366 examples pulled from eHRAF. Of these, nine examples did not describe aggressive mimicry, but contained specific references to cultural practices surrounding it. Of these nine, three (Andaman Islanders, Plains Omaha, and Amazonian Tukanos) had references to an absence of the practice or were ambiguous about its use. From the total 357 ethnographic examples, 145 cultures from 34 regions in all seven continental groups had some form of aggressive mimicry. This represents roughly 44% of eHRAF’s cultural dataset at the time of this search. Given the limited use of key terms excluding those in additional contexts, such as in the use of traps or snares where additional examples of aggressive mimicry may have been found, the breadth of our sample is representative of only a minimum percentage of societies that practiced aggressive mimicry, with the true sample size likely being larger than the 44% we identified.

The median unique forms of aggressive mimicry practiced by groups was three, with a range between one (*n* = 55, 38% of the sample) and ten (*n* = 1, < 1% of the total sample, among the Subarctic Ojibwa). Many texts contained references to the use of aggressive mimicry tactics employed against multiple animals but were only recorded once. For example, for the Yanomama of the Amazon and Orinoco Basin, the excerpt (Becher & Schütze, 1960) reads as follows: “They discover every [trail] of a wild animal, no matter how faint, sniff it, and announce the time when it was at this place. Moreover, they know how to attract the animals by imitative sounds.” Since the ethnographer only reported this tactic as being employed against “animals,” only one instance could be recorded.

These data are mostly intended to demarcate presence/absence, and very few (*n* = 2, < 1% of the total sample) mention an absence of the pattern. For example, among the Amazonian Tukanos, the use of mimicry is noted, but the ambiguousness of the excerpt did not allow us to record this group as using the mimicry for luring: “there they patiently stay in ambush without making any noise, or imitating the chirping of the birds that they want to kill. . . . When they hunt with a companion, imitations of bird calls are used for communication” (Jackson, 1983). The ethnographer may have intended to say that the hunters sit in silence or that, while waiting for birds, they imitate bird calls, but the ambiguity of the sentence and lack of context clues did not allow us to determine whether a call was being used for deception.

### All Forms of Sensory Exploitation for Each Culture Across Prey Types

With regard to the modes of sensory exploitation, results are reported in Table 1. 37% (*n* = 133) of all mimicry was acoustic, 32% (*n* = 115) was visual, 13% (*n* = 45) was baiting, 6% (*n* = 21) was fishing (whereby the sensory mode being exploited was ambiguous but directed toward fish), 6% (*n* = 21) was mixed (largely a mixture of visual/acoustic or visual/olfactory), 3% (*n* = 9) was fire fishing (employing fire to lure fish to the surface of the water), 2% (*n* = 9) was olfactory, 1% were ambiguous (*n* = 3), and 1% (*n* = 3) involved ground vibrations (limited to cases involving the luring of kangaroos or termites). Since many (but not all) forms of fishing involve the same type of general baiting method, the percentages reported in a modified sample (*n* = 283 of 357) with fishing removed are given in Table 2.

**Table 1:** Sensory exploitations for all forms of mimicry

Exploitation	*N*	%
Acoustic	133	37.3
Visual	115	32.2
Bait	45	12.6
Fishing	21	5.9
Mixed	21	5.9
Fire Fishing	9	2.5
Olfactory	7	2.0
Vibration	3	0.8
Unknown	3	0.8

**Table 2:** Sensory exploitation for non-fishing forms of mimicry

Exploitation	*N*	%
Acoustic	124	43.8
Visual	87	30.7
Bait	40	14.1
Mixed	20	7.1
Olfactory	7	2.5
Vibration	3	1.1
Unknown	2	0.7

A number of forms of fish exploitation did not involve direct line baiting. Examples include the use of shark rattles among the Melanesian Trobrianders and Santa Cruz Islanders and Polynesian Samoans; the construction of rat dummies among Samoans and Polynesian Tongans; the luring of fish through the trapping of conspecifics by Tongans, Samoans, and North American Northern Paiutes; and the use of other acoustic signals by East African Nuer, Micronesian Woleains, Polynesian Samoans and Lau Fijians, and Amazonian Ticuna. We found 53 examples of non-line-baiting forms of fish sensory exploitations. 52% of these (*n* = 28) were visual, 17% (*n* = 9) were acoustic, 17% (*n* = 9) were fire fishing, 9% were baiting (*n* = 5), 2% (*n* = 1) were ambiguous, and 2% (*n* = 1) were mixed. “Baiting” refers to the use of a fish’s conspecifics and “Fire Fishing” refers to the use of torches for luring fish to the surface at night.

### Breakdown of Sensory Exploitation by Subsistence Type

Besides geographic continent and region, the eHRAF database breaks down each culture by its subsistence type. In this search, eight subsistence types were identified, representing different proportions of the entire sample: Agro-Pastoralists (*n* = 11, 3% of all examples), Commercial Economy (*n* = 1, < 1%), Horticulturalists (*n* = 61, 17%), Hunter-Gatherers (*n* = 155, 43%), Intensive Agriculturalists (*n* = 26, 7%), Other Subsistence Combinations (*n* = 58, 16%), Pastoralists (*n* = 17, 5%), and Primarily Hunter-Gatherers (*n* = 28, 8%). To simplify the analysis, we broke the categories down into three groups loosely based on the sociopolitical typology of complexity described by Service (1963): foraging societies, horticultural societies, and pastoral and agricultural societies. Since the social systems of pastoralists and agriculturalists are not necessarily dissimilar in terms of scale, we collapsed these two categories into one. We keep a distinction between horticulturalists and this group because they represent a subsistence state combining a primary reliance on foraged foods and a primary reliance on agricultural products. This classification yielded the following three groups: hunter-gatherers (*n* = 183, 51%), horticulturalists (*n* = 61, 17%), and other subsistence types (*n* = 113, 31%). A breakdown of sensory exploitations for each of these groups can be found in Table 3.

**Table 3:** Sensory exploitation by subsistence type

Subsistence Type	Exploitation	*N*	%
Hunter-Gatherers	Visual	68	37.2
	Acoustic	65	35.5
	Bait	24	13.1
	Mixed	10	5.5
	Fishing	7	3.8
	Olfactory	5	2.7
	Unknown	2	1.1
	Fire Fishing	2	1.1
	Total		100.0
Horticulturalists	Acoustic	35	57.4
	Visual	10	16.4
	Bait	5	8.2
	Mixed	5	8.2
	Fishing	4	6.6
	Vibration	2	3.3
	Total		100.0
Other Combinations	Visual	37	32.7
	Acoustic	33	29.2
	Bait	16	14.2
	Fishing	10	8.8
	Fire Fishing	7	6.2
	Mixed	6	5.3
	Olfactory	2	1.8
	Unknown	1	0.9
	Vibration	1	0.9
	Total		100.0

### Representativeness of Each Subsistence Type, Presence of Aggressive Mimicry, and Overall Number of Cultures of a Specific Type in the eHRAF Dataset

To what extent are the samples from our search representative of eHRAF’s entire dataset more broadly? For cultures within our eHRAF search which were categorized as hunter-gatherers, the sample consists of exclusive hunter-gatherers (51 cultures) and *primarily* hunter-gatherers (14 cultures). The eHRAF database contained 58 hunter-gatherer groups, meaning 51/58 (88%) had some form of aggressive mimicry. For eHRAF’s “primarily hunter-gatherer” units, 14/26 (54%) had aggressive mimicry. For horticulturalists, roughly half of the examples had some form of aggressive mimicry (49%, 26/53), and 29% (54/189) of the remaining societies have examples of aggressive mimicry. Values for the representativeness of each subsistence type compared with the total number of societies of that subsistence type contained in eHRAF at the time of our search are provided in Table 4.

**Table 4:** Representativeness of sample by subsistence type and number displaying aggressive mimicry

Subsistence Type	Appeared in Search	Displays Aggressive Mimicry	Total eHRAF Cultures
Hunter-gatherer	52	51	58
Primarily hunter-gatherer	16	14	26
Horticulturalists	27	26	53
Other combinations	55	54	189
Total	150	145	326

### Directionality of Each Form of Mimicry

Finally, the directionality of each incident of aggressive mimicry was recorded for this study, yielding the following values: direct, indirect, fishing, and ambiguous. The results of these are in Table 5. “Direct” refers to a hunter luring an animal directly to him/herself, rather than toward an external object, scent, sound, or decoy (denoted as indirect). The overall results are as follows: 54% (*n* = 191) direct, 45% (*n* = 161) indirect, 1% (*n* = 4) did not specify, and < 1% (*n* = 1) ambiguous.

**Table 5:** Directionality of mimicry

Directionality	*N*	%
Direct	191	53.5
Indirect	161	45.1
Unknown	4	1.1
N/A	1	0.3

## Discussion

Our reviews of the animal literature and ethnographic record suggest that although cognitive aggressive mimicry is rare among animals, it is ubiquitous among human hunters. An exploration of the role of aggressive mimicry in the human niche might inform long-standing debates regarding our cognitive evolution. While there is fair agreement that the human mind is and has been shaped through its interaction with the minds of other humans, be it through evolutionary, developmental, or cultural processes, much less is known about how it has been and continues to be shaped by the minds of nonhuman agents.

Following our discussion of the Social Brain Hypothesis and the Foraging Brain Hypothesis, we propose that the use of deception in hunting contexts could serve as a link between the two theories’ different points of emphasis. The Social Brain Hypothesis explains the intraspecific adaptive value of each of our cognitive traits, whereas the Foraging Brain Hypothesis explains many of these traits in terms of external environmental problems that early humans had to solve. The gap between the points of emphasis separating these two theories may be fertile grounds for theoretical exploration. As noted by Kaplan et al. (2000:177), “It seems likely that the cognitive ability associated with foraging in a complex three-dimensional environment is an important pre-adaptation for social intelligence and complex social relations.” By linking the use of these intraspecific traits to interactions with prey in order to acquire food, we provide one plausible link between foraging cognition and social cognition. A possible pathway for these traits follows.

As the Pleistocene environment began fluctuating, humans were driven into a novel dietary niche characterized by dietary plasticity and an emphasis on hard-to-obtain foods that other animals (such as competing baboons) were not focused on. Following this came adaptations for behavioral plasticity which necessitated (1) more expensive and larger brains and (2) longer lifespans to afford such expenses, as in the Foraging Brain Hypothesis (Kaplan et al., 2000). From this expensive brain came more plastic behaviors and domain-general cognition, providing humans with a “cognitive buffer” against less predictable environments (Sol, 2009), including the ability to copy the successful behaviors of others (Boyd & Richerson, 1996; Muthukrishna et al., 2018) and the deception of prey. These traits were then employed in a social setting, leading to classic forms of Machiavellian deception and language that we find associated with the Social Brain Hypothesis (Barton & Dunbar, 1997). Several of these traits, including Theory of Mind and deception, language, and empathy, are discussed further below.

### The Evolution of Human Deception

In the ethnographic accounts of aggressive mimicry, two interrelated concepts are at play for human–animal relationships: Theory of Mind and its use for deception. Theory of Mind, or the recognition that other animals have mental states themselves, is often invoked in accounts of Machiavellian intelligence, whereby Theory of Mind and other facets of human cognition arose through an evolutionary arms race. As group sizes increased and more complex patterns of group behavior emerged, those who were better at manipulating and deceiving others might have secured more resources for themselves (Dunbar, 1998; Whiten & Byrne, 1988a). However, deception toward conspecifics is rare, largely for the reason that as deceptive signals become more common, receivers are selected to become less attuned to them or evolve better detection mechanisms. This causes the benefits of transmitting deceptive signals to decrease. Hence, most communication between *conspecifics* tends to be cooperative and honest (Dawkins & Krebs, 1978; Wallace, 1973).

But outside of interactions with minds with whom we cooperate and the evolutionary pressures toward honest signaling are interactions with minds with whom we do not regularly interact, as in the case of our prey. While it is undoubtedly true that humans lie to each other, and do so extensively, in our early evolutionary history prior to complex communication, our use of deception may have been primarily geared toward non-conspecifics (Barrett, 1999). Early forms of deception toward prey may have scaffolded further elaboration for deception against other humans. For example, patterns observed among 10 hunter-gatherer societies in the Probability Sample Files within HRAF show that most hunter-gatherer disguises in these groups were primarily used in the context of hunting, rather than being used for war (Buckner, 2021). The use of deception and the application of Theory of Mind toward other animals may have guided further forms of deception and fictioneering in general, such as in early animistic religions where ascriptions of agency are not limited to humans but extends to nonhuman animals and inanimate objects (Willerslev, 2007).

From a paleoanthropological standpoint, this cognitive suite of human mental capacities likely arose at the same time that early humans underwent their shift into a unique dietary niche, switching from lower-nutrient, easier-to-obtain foods to higher-nutrient, more-difficult-to-obtain foods, such as meat from other animals (Kaplan et al., 2000). During this period when humans began to explore a carnivorous niche, an increased consumption of fats and proteins allowed the evolution of a larger human brain and simultaneously the development of behavioral strategies to continue to feed itself (Bunn & Gurtov, 2014). Among these behavioral strategies was the suite of cognitive traits we have come to define as the human mental niche (Kaplan et al., 2000; Muthukrishna et al., 2018).

Recent evidence points to the idea that our earliest ancestors at early hominin kill sites were ambush predators (Bunn & Gurtov, 2014). The use of aggressive mimicry in these contexts to lure prey therefore may have scaffolded further brain evolution and the movement of the genus *Homo* into a carnivore niche, subsequently similarly scaffolding the use of these mental traits in nonhunting contexts. Take, for example, Theory of Mind, or the ability to ascribe mental states in others, as in perspective-taking, mental-state attribution, or desire attribution (Krupenye & Call, 2019). Several examples from the literature compiled here explicitly reflect an understanding of animals’ mindsets during deception. These range from more straightforward attributions of mental states, such as among the Guaraúno, where “to trap. . . means the same as to deceive the fish. . . spearing the fish by fooling him” (Turrado & Muirden 1945), to including explicit forms of perspective-taking, as among the Yukaghir, where one hunter notes, “You need to think like this: ‘What would attract my curiosity as a sable?’ This is how you need to think” (Willerslev, 2007:91), to attributions of complex, human-like mental states to animals and their spirits. Complex attributions are illustrated by those noted by Dentan (1968) among the Semai: “The east Semai do not use the real ‘name’ of an animal they are hunting or eating. Instead of defying something that threatens them, the Semai try to deceive it,” and Jenness (1935) among the Ojibwa, “Animals are subject to deception no less than human beings, and the shadow of the deer (or moose) will be thrown off its guard, will believe that you are not engaged in hunting, and will fail to carry back a warning.”

### The Evolution of Vocal Plasticity

One of the few aspects that separate human language ability from primate vocal abilities is its flexibility and plasticity. To this extent, Cheney and Seyfarth (2005) have argued that primate brains are almost primed for language perception. In primates, a limited number of signals can be perceived and employed in an almost limitless number of contexts. Nor is this unique to primates. As noted by Cheney and Seyfarth (2005:142), “while the number of distinct calls that animals produce is highly constrained, the number of signs that a parrot, dolphin, sea lion, or chimpanzee can learn to associate with a given stimulus or outcome is, if not limitless, certainly in the tens to hundreds.” As shown in ape language experiments, the issue with human language is not in its perception, but in its production (Cheney & Seyfarth, 1998; Fitch, 2011). A central remaining question for Seyfarth and Cheney (2010:97) then is, “Why should an individual who can deduce an almost limitless number of meanings from the calls of others be able to produce only a limited number of calls of his or her own?” Their answer is that Theory of Mind came first, and plasticity came later. Yet such an answer does not give any indication as to what the early forms of vocal plasticity may have looked like or why sociality and Theory of Mind would be so important.

What might such early forms of plasticity look like? For humans, the use of vocal aggressive mimicry is one potential substrate (Knight & Lewis, 2017). For all regions save for Europe and North America, acoustic exploitations were the primary form of aggressive mimicry, and among acoustic mimicry, 85.6% of it was as a direct (self-emitted) imitation of prey or predators of prey. Not only does aggressive mimicry provide a causal ecological driver for the evolution of vocal plasticity, it also at least partially provides a causal driver for Theory of Mind. As noted by Schultze (1907:496–98), while working among the Khoi,

Here it is especially clear how the choice of the words, their sequence and accentuation, aim at an imitation of the animal voice. It seems to me that certain observations from the primitive stages of an incipient literature (such as the Hottentots represent) are not without value in determining how man originally came to give his speeches certain rhythms when he wants to free them from oppressive monotony and use them for freer creations of the phantasy. It is no hypothesis but an ascertainment of the actual state of affairs that, in the Nama language, the childish joy in imitating certain animal voices with words represents one way to rhythmical development.

Such an idea was perhaps first elaborated by Lucretius in his sole surviving work, *De rerum natura*, written in the middle of the first century BC: “Men learnt to mimic with their mouths the trilling notes of birds long before they were able to enchant the ear by joining together in tuneful song” (Lucretius, 1988:213).

This hypothesis finds support in recent developments in phonetic theory. Perception-for-Action-Control Theory (PACT) posits that, based on neurological evidence showing that humans actively take perceived sounds from the environment and develop them into pre-motor action, our acoustic systems were adapted for pre-linguistic functions involving mimicry (Schwartz et al., 2007, 2012). As Schwartz et al. (2007:114) argue, “PACT assumes that speech perception not only allows listeners to follow the vocalizations. . . in order to understand them, but also to imitate and learn.” While nonhuman primates can technically produce the same range of phonetic sounds that humans can, the strengthening of neuro-motor connections between our perceptual systems and our vocal apparatus would have given us, and currently provides modern hunters with, the vocal breadth to mimic virtually any prey item through the copying of complex calls, trills, and whistles, and may have scaffolded the further development of language with its own vast but seemingly arbitrary phonetic breadth.

### The Evolution of Empathy

Why do people love animals but also want to eat them? Such a question is posited as an enigma in psychology, where it has been given the appropriate name, “The Meat Paradox” (Loughnan et al., 2010). In the ethnographic reports gathered from eHRAF, a number of records explicitly note feelings of regret and sympathy by hunters toward their prey. As stated by one informant among the Semai of the Malay Peninsula, “You have to deceive and trap your food, but you know that it is a bad thing to do, and you don’t want to do it” (Dentan, 2008). In a similar manner, Willerslev (2007:78) reports a Siberian Yukaghir hunter stating, “When killing an elk or a bear, I sometimes feel I’ve killed someone human. But one must banish such thoughts or one would go mad from shame.”

Despite the lethality involved, hunters often express a desire to not offend, or to show special respect and courtesy toward, their prey. Among both the Guayaki of the Amazon and the Batek of Malaysia, the common names of animals are often not used in association with the hunt since this is considered disrespectful to the animal. Offerings of food or drink, special methods of treating and disposing of the carcass, and other rituals of honor that impose some cost on the hunter are commonly found across many hunting societies. There is an oft-reported notion that the materials and sustenance provided by the prey animal must be remitted by the hunter, through particular demonstrations of appreciation, to appease the animal spirits and propitiate their continued presence in the world (Halfon & Barkai, 2020).

Although the employment of emotional empathy towards our prey seemingly constitutes a paradox, the proposed evolutionary link between empathy and Theory of Mind may yield a productive avenue for elucidating its origins (Seyfarth & Cheney, 2013). Consider the case in which Theory of Mind is applied to animals to better understand or predict their behavior (just as it is applied to other humans): the recognition that an animal or other organism shares a mind like one’s own immediately opens lines of empathy from human to animal (Shepard, 1998; Willerslev, 2004). Paradoxically, indeed, our ability to recognize the mental worlds of our prey allows us to better predict their behavior in the future while tragically allowing us to understand, or even inaccurately project, our own feelings regarding their demise.

## Conclusion

In conclusion, cognitive aggressive mimicry appears to be rare in nonhumans, but ubiquitous in humans. Hunters around the world, in subsistence-level societies and in developed countries where they pursue game for sport, employ a range of deceptive practices and mimicry aimed toward their prey. This deception can take the form of directly copying the calls of animals with our own vocal apparatus, luring animals in with hurt conspecifics, baiting them with potential mates or competitors, and creating completely false decoys which faithfully deceive them. Whether this arose early or late in our evolutionary trajectory is uncertain, but the use of it in virtually every hunter-gatherer society in the Human Relations Area Files database indicates that it would have yielded serious fitness benefits for early humans who were able to lure prey.

Although this pattern is extensively found around the world, its evolutionary and cognitive implications are varied. Further research specifically on Theory of Mind, vocal mimicry of prey, and empathy toward prey may help elucidate what role this practice may have played in our evolutionary history since our preliminary search indicates that the presence of aggressive mimicry early in our evolutionary past may partially explain several long-standing questions in these areas.

Finally, we hope that the role that nonhuman minds play in shaping human minds is given more attention in future evolutionary research. We recognize that mimicry and deception are not the full extent to which animal–human interactions may have played a role in developing our psyche, and that forms of cooperation and mutualisms likely also played a role (Cram et al., 2022). Take for example the interaction between honeyguides and the Hadza of East Africa, where Hadza foragers use whistles to call small birds which then lead them to honey. The coevolutionary relationship between hunters and birds is characterized by the manipulations of hunters, who destroy sources of honey to increase the hunger motivation of the honeyguides, and the benefits received by both parties in a process described as “manipulative mutualism” (Connor, 1995; Wood et al., 2014). Such complex relationships have not previously been well described in neither human nor broader evolutionary contexts. We suggest that future research should therefore continue to explore the natural history of human–animal interactions observed in the field. Additional questions elucidating our own hypothesis may explore what human and nonhuman interactions in the absence of Theory of Mind look like, as with cross-cultural development in children or the case of Temple Grandin, a researcher self-reportedly lacking Theory of Mind who has played an extensive role in promoting animal welfare around the world (Grandin, 1992).

### Electronic Supplementary Material

Below is the link to the electronic supplementary material.

Supplementary Material 1

## Data Availability

All data generated or analyzed during this study are included in the Zenodo repository, 10.5281/zenodo.6979028.
