# Supplementary material for: Aggressive Mimicry and the Evolution of the Human Cognitive Niche
Source: Hum Nat. 2023 Sep 6;34(3):456–75. doi: 10.1007/s12110-023-09458-y (PMC10543935; doi:10.1007/s12110-023-09458-y)
Supplement: Supplementary file 1 — Supplementary Material 1 [file 12110_2023_9458_MOESM1_ESM.pdf]

Electronic Supplementary Material for

Aggressive Mimicry and the Evolution of the Human Cognitive Niche

Cody Moser[1], William Buckner[2], Melina Sarian[3], Jeffrey Winking[4]

[1] Department of Cognitive and Information Sciences, University of California, Merced, Merced, CA, USA
[2] Human Systems and Behavior Lab, Department of Anthropology, Boston University, Boston, MA, USA
[3] Department of Anthropology, University of California, Davis, Davis, CA, USA
[4] Department of Anthropology, Texas A&M University, College Station, TX, USA

Correspondence: Cody Moser, cmoser2@ucmerced.edu

Human Nature 34(3), 2023, doi: https://doi.org/10.1007/s12110-023-09458-y

Culture	Continent	Region	Subsistence Type	Mimicry	Type of Lure	Exploitation	Lure Context	Type of Meat	Keyword
Ainu	Asia	East Asia	Hunter-gatherers	Yes	Direct	Acoustic	?	Deer	Decoy*
Ainu	Asia	East Asia	Hunter-gatherers	Yes	Indirect	Bait	Group	Deer	Lure* Luring
Ainu	Asia	East Asia	Hunter-gatherers	Yes	Indirect	Visual	Individual	Fish	Decoy*
Akan	Africa	Western Africa	Horticulturalists	Yes	Direct	Acoustic	Individual	Deer	Imitat*
Akan	Africa	Western Africa	Horticulturalists	Yes	Fishing	Fishing	Individual	Fish	Lure* Luring
Aleut	North America	Arctic and Subarctic	Hunter-gatherers	Yes	Direct	Acoustic	Individual	Seals	Lure* Luring
Aleut	North America	Arctic and Subarctic	Hunter-gatherers	Yes	Indirect	Mixed	Individual	Seals	Decoy*
Alutiiq	North America	Arctic and Subarctic	Hunter-gatherers	Yes	Direct	Acoustic	Individual	Deer	Decoy*
Alutiiq	North America	Arctic and Subarctic	Hunter-gatherers	Yes	Direct	Acoustic	Individual	Birds	Decoy*
Alutiiq	North America	Arctic and Subarctic	Hunter-gatherers	Yes	Indirect	Bait	Individual	Fish	Lure* Luring
Alutiiq	North America	Arctic and Subarctic	Hunter-gatherers	Yes	Indirect	Mixed	Individual	Seals	Decoy*
Alutiiq	North America	Arctic and Subarctic	Hunter-gatherers	Yes	Direct	Visual	Individual	Seals	Decoy*
Andamans	Asia	South Asia	Hunter-gatherers	No	N/A	N/A	N/A	N/A	Decoy*
Aranda	Oceania	Australia	Hunter-gatherers	Yes	Direct	Acoustic	Individual	Birds	Imitat*
Aranda	Oceania	Australia	Hunter-gatherers	Yes	Direct	Acoustic	Individual	Dingos	Imitat*
Aranda	Oceania	Australia	Hunter-gatherers	Yes	Indirect	Visual	Group	Kangaroos	Decoy*
Assiniboine	North America	Plains and Plateau	Hunter-gatherers	Yes	Direct	Acoustic	Group	Deer	Decoy*
Assiniboine	North America	Plains and Plateau	Hunter-gatherers	Yes	Direct	Mixed	Group	Buffalo	Decoy*
Assiniboine	North America	Plains and Plateau	Hunter-gatherers	Yes	Direct	Visual	Group	Antelope	Decoy*
Assiniboine	North America	Plains and Plateau	Hunter-gatherers	Yes	Direct	Visual	Group	Buffalo	Lure* Luring
Aymara	South America	Central Andes	Horticulturalists	Yes	Direct	Acoustic	?	Ducks	Lure* Luring
Azande	Africa	Central Africa	Horticulturalists	Yes	Direct	Vibration	Group	Termites	Deceive*
Azande	Africa	Central Africa	Horticulturalists	Yes	Indirect	Visual	Individual	Jackals	Deceive*
Aztecs	Middle America and Caribbean	Central Mexico	Intensive Agriculturalists	Yes	Indirect	Visual	Individual	Ducks	Lure* Luring
Banyoro	Africa	Eastern Africa	Horticulturalists	Yes	Direct	Acoustic	Individual	Predators	Imitat*
Bedouin	Middle East	Middle East	Pastoralists	Yes	Indirect	Visual	Individual	Birds	Decoy*
Bemba	Africa	Southern Africa	Horticulturalists	Yes	Direct	Acoustic	Individual	Duiker	Imitat*
Bemba	Africa	Southern Africa	Horticulturalists	Yes	Direct	Acoustic	Individual	Duiker	Imitat*
Blackfoot	North America	Plains and Plateau	Hunter-gatherers	Yes	Direct	Acoustic	Group	Buffalo	Lure* Luring
Blackfoot	North America	Plains and Plateau	Hunter-gatherers	Yes	Direct	Visual	Group	Buffalo	Decoy*
Blackfoot	North America	Plains and Plateau	Hunter-gatherers	Yes	Direct	Visual	Group	Antelope	Decoy*
Blackfoot	North America	Plains and Plateau	Hunter-gatherers	Yes	Direct	Visual	Group	Buffalo	Lure* Luring
Bororo	South America	Eastern South America	Hunter-gatherers	Yes	Direct	Acoustic	?	?	Mimic*
Botocudo	South America	Eastern South America	Hunter-gatherers	Yes	Direct	Acoustic	Individual	?	Imitat*
Burmans	Asia	Southeast Asia	Intensive Agriculturalists	Yes	Direct	Acoustic	Group	Deer	Deceive*
Burmans	Asia	Southeast Asia	Intensive Agriculturalists	Yes	Indirect	Bait	Group	Elephants	Decoy*
Burmans	Asia	Southeast Asia	Intensive Agriculturalists	Yes	Indirect	Mixed	Group	Birds	Lure* Luring
Cajuns	North America	Regional and Ethnic Cultures	Other Subsistence Combinations	Yes	Indirect	?	Individual	Conch larvae	Lure* Luring
Cajuns	North America	Regional and Ethnic Cultures	Other Subsistence Combinations	Yes	Indirect	Visual	Individual	Birds	Decoy*
Cambodians	Asia	Southeast Asia	Intensive Agriculturalists	Yes	Indirect	Mixed	Individual	Birds	Lure* Luring
Canela	South America	Eastern South America	Hunter-gatherers	Yes	Indirect	Bait	Individual	Parrots	Lure* Luring
Cherokee	North America	Eastern Woodlands	Other Subsistence Combinations	Yes	Direct	Acoustic	Individual	Turkeys	Decoy*
Cherokee	North America	Eastern Woodlands	Other Subsistence Combinations	Yes	Direct	Acoustic	?	Deer	Decoy*
Cherokee	North America	Eastern Woodlands	Other Subsistence Combinations	Yes	Direct	Acoustic	Individual	Birds	Imitat*
Cherokee	North America	Eastern Woodlands	Other Subsistence Combinations	Yes	Indirect	Bait	Individual	Fish	Lure* Luring
Cherokee	North America	Eastern Woodlands	Other Subsistence Combinations	Yes	Indirect	Bait	Individual	Deer	Lure* Luring
Cherokee	North America	Eastern Woodlands	Other Subsistence Combinations	Yes	Direct	Visual	Individual	Deer	Decoy*
Cherokee	North America	Eastern Woodlands	Other Subsistence Combinations	Yes	Direct	Visual	Individual	Buffalo	Decoy*
Cherokee	North America	Eastern Woodlands	Other Subsistence Combinations	Yes	Direct	Visual	Individual	Bears	Decoy*
Cherokee	North America	Eastern Woodlands	Other Subsistence Combinations	Yes	Direct	Visual	Individual	Deer	Lure* Luring
Chinookans	North America	Northwest Coast and California	Hunter-gatherers	Yes	Direct	Acoustic	?	Elk	Decoy*
Chinookans	North America	Northwest Coast and California	Hunter-gatherers	Yes	?	Visual	?	Elk	Decoy*
Chinookans	North America	Northwest Coast and California	Hunter-gatherers	Yes	Indirect	Visual	Individual	Fish	Lure* Luring
Chinookans	North America	Northwest Coast and California	Hunter-gatherers	Yes	Indirect	Visual	Individual	Deer	Lure* Luring
Chipewyans	North America	Arctic and Subarctic	Hunter-gatherers	Yes	Direct	Acoustic	Individual	Moose	Decoy*
Chipewyans	North America	Arctic and Subarctic	Hunter-gatherers	Yes	Direct	Acoustic	Individual	Caribou	Decoy*
Chipewyans	North America	Arctic and Subarctic	Hunter-gatherers	Yes	Indirect	Bait	Individual	Beavers	Lure* Luring
Chipewyans	North America	Arctic and Subarctic	Hunter-gatherers	Yes	Indirect	Visual	Individual	Foxes	Decoy*
Chorote	South America	Southern South America	Hunter-gatherers	Yes	Direct	Acoustic	Individual	?	Decoy*

Chukchee	Asia	North Asia	Pastoralists	Yes	Indirect	Olfactory	Group	Reindeer	Decoy*
Chukchee	Asia	North Asia	Pastoralists	Yes	Direct	Visual	Individual	Seals	Imitat*
Chukchee	Asia	North Asia	Pastoralists	Yes	Direct	Visual	Individual	Reindeer	Lure* Luring
Chukchee	Asia	North Asia	Pastoralists	Yes	Direct	Visual	Individual	Fish	Lure* Luring
Chuuk	Oceania	Micronesia	Other Subsistence Combinations	Yes	Indirect	Fishing	Individual	Fish	Lure* Luring
Chuuk	Oceania	Micronesia	Other Subsistence Combinations	Yes	Direct	Visual	?	Fish	Lure* Luring
Chuuk	Oceania	Micronesia	Other Subsistence Combinations	Yes	Indirect	Visual	Individual	Fish	Deceive*
Comanche	North America	Plains and Plateau	Hunter-gatherers	Yes	Indirect	Mixed	Individual	Horses	Lure* Luring
Comanche	North America	Plains and Plateau	Hunter-gatherers	Yes	Direct	Visual	Group	Buffalo	Decoy*
Copper Inuit	North America	Arctic and Subarctic	Hunter-gatherers	Yes	Indirect	Acoustic	Individual	Foxes	Lure* Luring
Copper Inuit	North America	Arctic and Subarctic	Hunter-gatherers	Yes	Indirect	Fishing	Individual	Fish	Lure* Luring
Copper Inuit	North America	Arctic and Subarctic	Hunter-gatherers	Yes	Direct	Visual	Group	Deer	Imitat*
Copper Inuit	North America	Arctic and Subarctic	Hunter-gatherers	Yes	Direct	Visual	Group	Caribou	Lure* Luring
Copper Inuit	North America	Arctic and Subarctic	Hunter-gatherers	Yes	Indirect	Visual	Individual	Fish	Lure* Luring
Creek	North America	Eastern Woodlands	Primarily Hunter-gatherers	Yes	Direct	Acoustic	Individual	Deer	Imitat*
Crow	North America	Plains and Plateau	Hunter-gatherers	Yes	Direct	Acoustic	Group	Buffalo	Lure* Luring
Delaware	North America	Eastern Woodlands	Primarily Hunter-gatherers	Yes	Direct	Acoustic	Individual	Deer	Imitat*
Delaware	North America	Eastern Woodlands	Primarily Hunter-gatherers	Yes	Indirect	Bait	Individual	Otters	Lure* Luring
Delaware	North America	Eastern Woodlands	Primarily Hunter-gatherers	Yes	Indirect	Olfactory	Individual	Beavers	Decoy*
Dogon	Africa	Southern Africa	Horticulturalists	Yes	Direct	Acoustic	Individual	Birds	Imitat*
Dogon	Africa	Western Africa	Intensive Agriculturalists	Yes	Direct	Acoustic	Individual	Mice	Lure* Luring
Eastern Apache	North America	Southwest and Basin	Hunter-gatherers	Yes	Direct	Visual	Individual	Deer	Imitat*
Eastern Apache	North America	Southwest and Basin	Hunter-gatherers	Yes	Direct	Visual	Individual	Antelope	Imitat*
Eastern Toraja	Asia	Southeast Asia	Horticulturalists	Yes	Direct	Acoustic	Individual	Birds	Decoy*
Eastern Toraja	Asia	Southeast Asia	Horticulturalists	Yes	Indirect	Bait	Group	Buffalo	Decoy*
Eastern Toraja	Asia	Southeast Asia	Horticulturalists	Yes	Indirect	Bait	Individual	Birds	Decoy*
Eastern Toraja	Asia	Southeast Asia	Horticulturalists	Yes	Indirect	Mixed	Individual	Birds	Lure* Luring
Eastern Toraja	Asia	Southeast Asia	Horticulturalists	Yes	Indirect	Mixed	Group	Birds	Lure* Luring
Eastern Toraja	Asia	Southeast Asia	Horticulturalists	Yes	Indirect	Mixed	Individual	Birds	Imitat*
Eastern Toraja	Asia	Southeast Asia	Horticulturalists	Yes	Direct	Vibration	Individual	Woodpeckers	Imitat*
Fox	North America	Eastern Woodlands	Primarily Hunter-gatherers	Yes	Indirect	Bait	Individual	Mink	Lure* Luring
Gros Ventre	North America	Plains and Plateau	Hunter-gatherers	Yes	Direct	Visual	Group	Buffalo	Decoy*
Guaraní	South America	Eastern South America	Other Subsistence Combinations	Yes	Direct	Acoustic	Individual	Parrots	Imitat*
Guaraní	South America	Eastern South America	Other Subsistence Combinations	Yes	Direct	Acoustic	Individual	Monkey	Imitat*
Haida	North America	Northwest Coast and California	Hunter-gatherers	Yes	Direct	Acoustic	Individual	Deer	Lure* Luring
Havasupai	North America	Southwest and Basin	Other Subsistence Combinations	Yes	Direct	Acoustic	Group	Deer	Lure* Luring
Havasupai	North America	Southwest and Basin	Other Subsistence Combinations	Yes	Direct	Visual	?	Antelope	Mimic*
Hawaiians	Oceania	Polynesia	Other Subsistence Combinations	Yes	Direct	Acoustic	Individual	Birds	Imitat*
Hawaiians	Oceania	Polynesia	Other Subsistence Combinations	Yes	Fishing	Fishing	Individual	Fish	Decoy*
Hawaiians	Oceania	Polynesia	Other Subsistence Combinations	Yes	Indirect	Visual	Individual	Fish	Lure* Luring
Hawaiians	Oceania	Polynesia	Other Subsistence Combinations	Yes	Indirect	Visual	Individual	Fish	Mimic*
Hopi	North America	Southwest and Basin	Intensive Agriculturalists	Yes	Direct	Acoustic	Individual	Deer	Decoy*
Hopi	North America	Southwest and Basin	Intensive Agriculturalists	Yes	Direct	Acoustic	Individual	Birds	Imitat*
Hopi	North America	Southwest and Basin	Intensive Agriculturalists	Yes	Indirect	Bait	Group	Birds	Lure* Luring
Huichol	Middle America and Caribbean	Northern Mexico	Other Subsistence Combinations	Yes	Direct	Acoustic	Group	Deer	Decoy*
Huichol	Middle America and Caribbean	Northern Mexico	Other Subsistence Combinations	Yes	Indirect	Bait	Individual	Birds	Decoy*
Ifugao	Asia	Southeast Asia	Intensive Agriculturalists	Yes	Direct	Acoustic	Individual	Birds	Imitat*
Ifugao	Asia	Southeast Asia	Intensive Agriculturalists	Yes	Indirect	Bait	Individual	Birds	Lure* Luring
Igbo	Africa	Western Africa	Horticulturalists	Yes	Direct	Acoustic	Individual	Mammals	Imitat*
Igbo	Africa	Western Africa	Horticulturalists	Yes	Direct	Acoustic	Individual	Birds	Imitat*
Imperial Romans	Europe	Southern Europe	Intensive Agriculturalists	Yes	Indirect	Acoustic	Individual	Birds	Decoy*
Ingalik	North America	Arctic and Subarctic	Hunter-gatherers	Yes	?	Visual	?	Caribou	Decoy*
Innu	North America	Arctic and Subarctic	Hunter-gatherers	Yes	Direct	Acoustic	Group	Mammals	Decoy*
Innu	North America	Arctic and Subarctic	Hunter-gatherers	Yes	Direct	Acoustic	Individual	Birds	Lure* Luring
Innu	North America	Arctic and Subarctic	Hunter-gatherers	Yes	Indirect	Visual	Group	Deer	Decoy*
Innu	North America	Arctic and Subarctic	Hunter-gatherers	Yes	Indirect	Visual	Individual	Fish	Lure* Luring
Iroquois	North America	Eastern Woodlands	Horticulturalists	Yes	Indirect	Bait	Individual	Birds	Lure* Luring
Island Carib	Middle America and Caribbean	Caribbean	Primarily Hunter-gatherers	Yes	Direct	Acoustic	?	Birds	Decoy*
Island Carib	Middle America and Caribbean	Caribbean	Hunter-gatherers	Yes	Indirect	Bait	Individual	Birds	Lure* Luring
Jivaro	South America	Amazon and Orinoco	Horticulturalists	Yes	Direct	Acoustic	Individual	Mammals	Decoy*

Jivaro	South America	Amazon and Orinoco	Horticulturalists	Yes	Direct	Acoustic	Individual	Birds	Imitat*
Jivaro	South America	Amazon and Orinoco	Horticulturalists	Yes	Direct	Acoustic	Individual	Monkey	Mimic*
Kapauku	Oceania	Melanesia	Intensive Agriculturalists	Yes	Direct	Acoustic	Individual	Pigs	Lure* Luring
Kapauku	Oceania	Melanesia	Intensive Agriculturalists	Yes	Indirect	Bait	Individual	Pigs	Lure* Luring
Kapauku	Oceania	Melanesia	Intensive Agriculturalists	Yes	Direct	Fire Fishing	Group	Fish	Lure* Luring
Karaja	South America	Eastern South America	Hunter-gatherers	Yes	Direct	Acoustic	?	Birds and Game	Lure* Luring
Karaja	South America	Eastern South America	Hunter-gatherers	Yes	Direct	Visual	?	Otters	Lure* Luring
Karen	Asia	Southeast Asia	Other Subsistence Combinations	Yes	Direct	Acoustic	Individual	Barking Deer	Imitat*
Karen	Asia	Southeast Asia	Other Subsistence Combinations	Yes	Direct	Acoustic	Individual	Birds	Imitat*
Karen	Asia	Southeast Asia	Other Subsistence Combinations	Yes	Indirect	Acoustic	Individual	Birds	Decoy*
Karen	Asia	Southeast Asia	Other Subsistence Combinations	Yes	Indirect	Acoustic	?	Mammals	Mimic*
Kaska	North America	Arctic and Subarctic	Hunter-gatherers	Yes	Direct	Acoustic	Individual	Moose	Imitat*
Kaska	North America	Arctic and Subarctic	Hunter-gatherers	Yes	Indirect	Olfactory	Individual	Predators	Lure* Luring
Kaska	North America	Arctic and Subarctic	Hunter-gatherers	Yes	Indirect	Visual	Individual	Caribou	Decoy*
Kaska	North America	Arctic and Subarctic	Hunter-gatherers	Yes	Indirect	Visual	?	Birds	Decoy*
Khasi	Asia	South Asia	Other Subsistence Combinations	Yes	Indirect	Bait	Individual	Birds	Lure* Luring
Khoi	Africa	Southern Africa	Other Subsistence Combinations	Yes	N/A	Acoustic	N/A	N/A	Imitat*
Kimam	Oceania	Melanesia	Intensive Agriculturalists	Yes	Direct	Vibration	Individual	Kangaroos	Imitat*
Kiribati	Oceania	Micronesia	Primarily Hunter-gatherers	Yes	Indirect	Fishing	Individual	Fish	Lure* Luring
Kiribati	Oceania	Micronesia	Primarily Hunter-gatherers	Yes	Indirect	Visual	Individual	Fish	Lure* Luring
Klamath	North America	Plains and Plateau	Hunter-gatherers	Yes	Direct	Acoustic	Individual/Group	Deer	Lure* Luring
Klamath	North America	Plains and Plateau	Hunter-gatherers	Yes	Direct	Visual	Individual/Group	Deer	Lure* Luring
Klamath	North America	Plains and Plateau	Hunter-gatherers	Yes	Direct	Visual	Individual	Birds/Fish	Lure* Luring
Korea	Asia	East Asia	Intensive Agriculturalists	Yes	Direct	Acoustic	Individual	Birds	Imitat*
Kuna	Middle America and Caribbean	Central America	Horticulturalists	Yes	Direct	Acoustic	Individual	Curassow	Imitat*
Kuna	Middle America and Caribbean	Central America	Horticulturalists	Yes	Indirect	Acoustic	Individual	Mammals	Lure* Luring
Kuna	Middle America and Caribbean	Central America	Horticulturalists	Yes	Indirect	Visual	Group	Turtles	Decoy*
Kutenai	North America	Plains and Plateau	Hunter-gatherers	Yes	Direct	Fishing	Individual	Fish	Lure* Luring
Kutenai	North America	Plains and Plateau	Hunter-gatherers	Yes	Indirect	Olfactory	Individual	Beavers	Lure* Luring
Kutenai	North America	Plains and Plateau	Hunter-gatherers	Yes	Indirect	Visual	?	Waterfowl	Decoy*
Lau Fijians	Oceania	Polynesia	Other Subsistence Combinations	Yes	Indirect	Acoustic	?	Fish	Lure* Luring
Lau Fijians	Oceania	Polynesia	Other Subsistence Combinations	Yes	Indirect	Visual	?	Fish	Lure* Luring
Lepcha	Asia	Central Asia	Intensive Agriculturalists	Yes	Direct	Acoustic	Individual	Mammals	Imitat*
Lepcha	Asia	Central Asia	Intensive Agriculturalists	Yes	Direct	Acoustic	Individual	Birds	Lure* Luring
Malays	Asia	Southeast Asia	Intensive Agriculturalists	Yes	Fishing	Fishing	Group	Fish	Lure* Luring
Manchu	Asia	East Asia	Intensive Agriculturalists	Yes	Direct	Mixed	Group	Deer	Lure* Luring
Maori	Oceania	Polynesia	Primarily Hunter-gatherers	Yes	Indirect	?	Individual	Birds	Lure* Luring
Maori	Oceania	Polynesia	Primarily Hunter-gatherers	Yes	Direct	Acoustic	Individual	Birds	Lure* Luring
Maori	Oceania	Polynesia	Primarily Hunter-gatherers	Yes	Indirect	Acoustic	Individual	Parrots	Decoy*
Maori	Oceania	Polynesia	Primarily Hunter-gatherers	Yes	Indirect	Bait	Individual	Birds	Lure* Luring
Maori	Oceania	Polynesia	Primarily Hunter-gatherers	Yes	Indirect	Visual	Individual	Birds	Decoy*
Maori	Oceania	Polynesia	Primarily Hunter-gatherers	Yes	Indirect	Visual	Individual	Fish	Lure* Luring
Maori	Oceania	Polynesia	Primarily Hunter-gatherers	Yes	Indirect	Visual	Individual	Birds	Lure* Luring
Maricopa	North America	Southwest and Basin	Primarily Hunter-gatherers	Yes	Direct	Visual	Individual	Deer	Imitat*
Marqueses	Oceania	Polynesia	Other Subsistence Combinations	Yes	Direct	Fire Fishing	Group	Fish	Lure* Luring
Marqueses	Oceania	Polynesia	Other Subsistence Combinations	Yes	Direct	Fishing	Group	Fish	Lure* Luring
Marshallese	Oceania	Micronesia	Other Subsistence Combinations	Yes	Indirect	Bait	Individual	Rats	Lure* Luring
Marshallese	Oceania	Micronesia	Other Subsistence Combinations	Yes	Direct	Fire Fishing	Individual	Fish	Lure* Luring
Marshallese	Oceania	Micronesia	Other Subsistence Combinations	Yes	Indirect	Visual	Individual	Fish	Imitat*
Mataco	South America	Southern South America	Hunter-gatherers	Yes	Direct	Acoustic	Individual	Parrots	Imitat*
Mataco	South America	Southern South America	Hunter-gatherers	Yes	Indirect	Bait	Group	Deer	Lure* Luring
Mataco	South America	Southern South America	Hunter-gatherers	Yes	Indirect	Bait	Individual	Birds	Lure* Luring
Maya	Middle America and Caribbean	Maya Area	Horticulturalists	Yes	Direct	Acoustic	Individual	Deer	Imitat*
Maya	Middle America and Caribbean	Maya Area	Horticulturalists	Yes	Direct	Acoustic	Individual	Birds	Imitat*
Mbuti	Africa	Central Africa	Hunter-gatherers	Yes	Direct	Acoustic	Group	Large Mammal	Lure* Luring
Mbuti	Africa	Central Africa	Hunter-gatherers	Yes	Direct	Acoustic	Individual	?	Imitat*
Mbuti	Africa	Central Africa	Hunter-gatherers	Contextual	N/A	N/A	N/A	N/A	Deceive*
Mende	Africa	Western Africa	Horticulturalists	Yes	Direct	Acoustic	Individual	Duiker	Decoy*
Mi'kmaq	North America	Eastern Woodlands	Hunter-gatherers	Yes	Direct	Acoustic	Individual	Moose	Lure* Luring
Mi'kmaq	North America	Eastern Woodlands	Hunter-gatherers	Yes	Direct	Fire Fishing	Individual	Fish	Lure* Luring

Mi'kmaq	North America	Eastern Woodlands	Hunter-gatherers	Yes	Indirect	Mixed	Individual	Waterfowl	Decoy*
Mi'kmaq	North America	Eastern Woodlands	Hunter-gatherers	Yes	Direct	Visual	Individual	Seals	Deception
Mi'kmaq	North America	Eastern Woodlands	Hunter-gatherers	Yes	Direct	Visual	Individual	Sea mammals	Decoy*
Mi'kmaq	North America	Eastern Woodlands	Hunter-gatherers	Yes	Indirect	Visual	Individual	Seals	Lure* Luring
Miskito	Middle America and Caribbean	Central America	Primarily Hunter-gatherers	Yes	Direct	Acoustic	Individual	Birds	Imitat*
Miskito	Middle America and Caribbean	Central America	Primarily Hunter-gatherers	Yes	Direct	Acoustic	Individual	Agouti	Lure* Luring
Miskito	Middle America and Caribbean	Central America	Primarily Hunter-gatherers	Yes	Indirect	Visual	?	Turtles	Decoy*
Mongo	Africa	Central Africa	Horticulturalists	Yes	Direct	Acoustic	Individual	Monkey	Decoy*
Mongo	Africa	Central Africa	Horticulturalists	Yes	Direct	Acoustic	Individual	Duiker, predators	Decoy*
Mongolia	Asia	Central Asia	Pastoralists	Yes	Fishing	Fire Fishing	Group	Fish	Lure* Luring
Mundurucu	South America	Amazon and Orinoco	Hunter-gatherers	Yes	Indirect	Mixed	Group	Tapir	Lure* Luring
Navajo	North America	Southwest and Basin	Agro-Pastoralists	Yes	Direct	Acoustic	Group	Deer	Imitat*
Navajo	North America	Southwest and Basin	Agro-Pastoralists	Yes	Indirect	Bait	Group	Eagles	Decoy*
Navajo	North America	Southwest and Basin	Agro-Pastoralists	Yes	Direct	Olfactory	Group	Deer	Imitat*
Navajo	North America	Southwest and Basin	Agro-Pastoralists	Yes	Indirect	Visual	Group	Birds/Eagles	Decoy*
Navajo	North America	Southwest and Basin	Agro-Pastoralists	Yes	Direct	Visual	Group	Deer	Imitat*
Navajo	North America	Southwest and Basin	Agro-Pastoralists	Yes	Indirect	Visual	Individual	Prairie dogs	Lure* Luring
Nenets	Asia	North Asia	Primarily Hunter-gatherers	Yes	Indirect	Bait	?	Reindeer	Decoy*
Nenets	Asia	North Asia	Primarily Hunter-gatherers	Yes	Indirect	Bait	?	Flies	Decoy*
Nicobarese	Asia	South Asia	Other Subsistence Combinations	Yes	Direct	Fire Fishing	Individual	Fish	Lure* Luring
Nivkh	Asia	North Asia	Primarily Hunter-gatherers	Yes	Direct	Visual	Individual	Wolves	Imitat*
Northern Paiute	North America	Southwest and Basin	Hunter-gatherers	Yes	Indirect	Bait	Individual	Fish	Decoy*
Northern Paiute	North America	Southwest and Basin	Hunter-gatherers	Yes	Direct	Visual	Group	Antelope	Deception
Northern Paiute	North America	Southwest and Basin	Hunter-gatherers	Yes	Indirect	Visual	Individual	Waterfowl	Decoy*
Northern Paiute	North America	Southwest and Basin	Hunter-gatherers	Yes	Indirect	Visual	Individual	Birds	Decoy*
Nuer	Africa	Eastern Africa	Agro-Pastoralists	Yes	Direct	Acoustic	Individual	Fish	Imitat*
Nuer	Africa	Eastern Africa	Agro-Pastoralists	Yes	Indirect	Acoustic	Individual	Fish	Imitat*
Nuu-chah-nulth	North America	Northwest Coast and California	Hunter-gatherers	Yes	Indirect	Visual	Individual	Fish	Decoy*
Nuxalk	North America	Northwest Coast and California	Hunter-gatherers	Yes	Indirect	Bait	Individual	Carnivores	Lure* Luring
Nuxalk	North America	Northwest Coast and California	Hunter-gatherers	Yes	Indirect	Fire Fishing	Individual	Fish	Lure* Luring
Ojibwa	North America	Arctic and Subarctic	Hunter-gatherers	Yes	Indirect	Bait	Individual	Birds	Decoy*
Ojibwa	North America	Arctic and Subarctic	Hunter-gatherers	Yes	Indirect	Bait	Individual	Predators	Lure* Luring
Ojibwa	North America	Arctic and Subarctic	Hunter-gatherers	Yes	Indirect	Bait	Individual	Deer	Lure* Luring
Ojibwa	North America	Arctic and Subarctic	Hunter-gatherers	Yes	Indirect	Fishing	Individual	Fish	Decoy*
Ojibwa	North America	Arctic and Subarctic	Hunter-gatherers	Contextual	N/A	N/A	N/A	N/A	Deception
Ojibwa	North America	Arctic and Subarctic	Hunter-gatherers	Yes	Direct	Visual	Individual	Mammals	Deceive*
Ojibwa	North America	Arctic and Subarctic	Hunter-gatherers	Yes	Direct	Visual	Group	Buffalo	Decoy*
Ojibwa	North America	Arctic and Subarctic	Hunter-gatherers	Yes	Indirect	Visual	Individual	Waterfowl	Decoy*
Ojibwa	North America	Arctic and Subarctic	Hunter-gatherers	Yes	Indirect	Visual	Individual	Fish	Decoy*
Ojibwa	North America	Arctic and Subarctic	Hunter-gatherers	Yes	Indirect	Visual	Individual	Birds	Lure* Luring
Okinawans	Asia	East Asia	Intensive Agriculturalists	Yes	Fishing	Visual	Individual	Cuttlefish	Lure* Luring
Omaha	North America	Plains and Plateau	Primarily Hunter-gatherers	No	N/A	N/A	N/A	N/A	Decoy*
Ona	South America	Southern South America	Hunter-gatherers	Yes	Direct	Acoustic	Individual	Birds	Deceive*
Ona	South America	Southern South America	Hunter-gatherers	Yes	Indirect	Mixed	Individual	Seals	Decoy*
O'odham	North America	Southwest and Basin	Other Subsistence Combinations	Yes	Direct	Mixed	Individual	Deer	Imitat*
Orokaiva	Oceania	Melanesia	Other Subsistence Combinations	Yes	Direct	Fishing	Individual	Fish	Lure* Luring
Ovimbundu	Africa	Southern Africa	Horticulturalists	Yes	Direct	Acoustic	Individual	Antelope	Decoy*
Ovimbundu	Africa	Southern Africa	Horticulturalists	Yes	Direct	Acoustic	?	Duiker	Lure* Luring
Ovimbundu	Africa	Southern Africa	Horticulturalists	Yes	Direct	Bait	?	Deer	Decoy*
Ovimbundu	Africa	Southern Africa	Horticulturalists	Contextual	N/A	N/A	N/A	N/A	Mimic*
Ovimbundu	Africa	Southern Africa	Horticulturalists	Yes	Direct	Visual	Group	Antelope	Decoy*
Pawnee	North America	Plains and Plateau	Primarily Hunter-gatherers	Yes	Direct	Visual	Group	Buffalo	Imitat*
Pomo	North America	Northwest Coast and California	Hunter-gatherers	Yes	?	?	Individual	Squirrels	Deception
Pomo	North America	Northwest Coast and California	Hunter-gatherers	Yes	Direct	Acoustic	Individual	Deer	Lure* Luring
Pomo	North America	Northwest Coast and California	Hunter-gatherers	Yes	Direct	Visual	Individual	Birds	Lure* Luring
Puerto Ricans	Middle America and Caribbean	Caribbean	Commerical Economy	Yes	Direct	Visual	Individual	Fish	Lure* Luring
Pumé	South America	Amazon and Orinoco	Other Subsistence Combinations	Yes	Direct	Visual	Individual	Jabiru Stork	Imitat*
Pumé	South America	Amazon and Orinoco	Other Subsistence Combinations	Yes	Direct	Visual	Individual	Deer	Mimic*
Quinault	North America	Northwest Coast and California	Hunter-gatherers	Yes	Direct	Acoustic	Individual	Deer	Lure* Luring
Quinault	North America	Northwest Coast and California	Hunter-gatherers	Yes	Indirect	Visual	Individual	Birds	Decoy*

Quinault	North America	Northwest Coast and California	Hunter-gatherers	Yes	Indirect	Visual	Individual	Fish	Lure* Luring
Rapa Nui	Oceania	Polynesia	Horticulturalists	Yes	Indirect	Fishing	Individual	Fish	Lure* Luring
Rural Irish	Europe	British Isles	Agro-Pastoralists	Yes	Direct	Visual	Individual	Fish	Lure* Luring
Rwandans	Africa	Central Africa	Agro-Pastoralists	Yes	Direct	Bait	Group	Elephants	Decoy*
Saami	Europe	Scandinavia	Pastoralists	Yes	Direct	Acoustic	Individual	Birds	Imitat*
Saami	Europe	Scandinavia	Pastoralists	Yes	Indirect	Bait	?	Reindeer	Decoy*
Saami	Europe	Scandinavia	Pastoralists	Yes	Indirect	Bait	Individual	Carnivores	Lure* Luring
Saami	Europe	Scandinavia	Pastoralists	Yes	Fishing	Fishing	Group	Fish	Lure* Luring
Saami	Europe	Scandinavia	Pastoralists	Yes	Indirect	Mixed	Group	Reindeer	Lure* Luring
Saami	Europe	Scandinavia	Pastoralists	Yes	Direct	Visual	?	Birds	Decoy*
Saami	Europe	Scandinavia	Pastoralists	Yes	Indirect	Visual	?	Birds	Decoy*
Saami	Europe	Scandinavia	Pastoralists	Yes	Direct	Visual	Group	Geese	Lure* Luring
Saami	Europe	Scandinavia	Pastoralists	Yes	Indirect	Visual	Individual	Beavers	Lure* Luring
Samoans	Oceania	Polynesia	Horticulturalists	Yes	Direct	Acoustic	Group	Sharks	Lure* Luring
Samoans	Oceania	Polynesia	Horticulturalists	Yes	Indirect	Acoustic	Individual	Fish	Decoy*
Samoans	Oceania	Polynesia	Horticulturalists	Yes	Indirect	Acoustic	Individual	Birds	Decoy*
Samoans	Oceania	Polynesia	Horticulturalists	Yes	Indirect	Bait	Individual	Fish	Lure* Luring
Samoans	Oceania	Polynesia	Horticulturalists	Yes	Indirect	Mixed	Individual	Fish	Decoy*
Samoans	Oceania	Polynesia	Horticulturalists	Yes	Indirect	Visual	Individual	Fish	Deceive*
Samoans	Oceania	Polynesia	Horticulturalists	Yes	Indirect	Visual	Individual	Fish	Deception
Samoans	Oceania	Polynesia	Horticulturalists	Yes	Indirect	Visual	Individual	Fish (squid)	Lure* Luring
Samoyed	Asia	North Asia	Primarily Hunter-gatherers	Yes	Indirect	Bait	Group	Reindeer	Decoy*
Samoyed	Asia	North Asia	Primarily Hunter-gatherers	Yes	Indirect	Visual	Group	Ducks	Decoy*
San	Africa	Southern Africa	Hunter-gatherers	Yes	Direct	Acoustic	Group	Duiker	Imitat*
San	Africa	Southern Africa	Hunter-gatherers	Yes	Direct	Acoustic	Individual	Bovids	Imitat*
San	Africa	Southern Africa	Hunter-gatherers	Yes	Direct	Acoustic	Individual	Wildebeest	Imitat*
San	Africa	Southern Africa	Hunter-gatherers	Yes	Indirect	Acoustic	Group	Kudu	Lure* Luring
San	Africa	Southern Africa	Hunter-gatherers	Yes	Direct	Visual	Individual	Termites	Lure* Luring
Santa Cruz Islanders	Oceania	Melanesia	Horticulturalists	Yes	Direct	Acoustic	Individual	Birds	Lure* Luring
Santa Cruz Islanders	Oceania	Melanesia	Horticulturalists	Yes	Indirect	Acoustic	Group	Sharks	Lure* Luring
Santa Cruz Islanders	Oceania	Melanesia	Horticulturalists	Yes	Indirect	Mixed	Individual	Birds	Decoy*
Santal	Asia	South Asia	Intensive Agriculturalists	Yes	Indirect	Mixed	Group	Birds	Decoy*
Semai	Asia	Southeast Asia	Horticulturalists	Contextual	N/A	N/A	N/A	N/A	Deceive*
Semai	Asia	Southeast Asia	Horticulturalists	Contextual	N/A	N/A	N/A	N/A	Deceive*
Semang	Asia	Southeast Asia	Hunter-gatherers	Yes	Direct	Acoustic	Individual	Birds	Deceive*
Semang	Asia	Southeast Asia	Hunter-gatherers	Yes	Direct	Acoustic	Individual	Gibbons	Imitat*
Semang	Asia	Southeast Asia	Hunter-gatherers	Yes	Direct	Acoustic	Individual	Monkey	Lure* Luring
Semang	Asia	Southeast Asia	Hunter-gatherers	Yes	Direct	Acoustic	Individual	Birds	Mimic*
Semang	Asia	Southeast Asia	Hunter-gatherers	Yes	Direct	Olfactory	Individual	Gibbons	Imitat*
Seminole	North America	Eastern Woodlands	Horticulturalists	Yes	Direct	Acoustic	Individual	Alligator	Imitat*
Seminole	North America	Eastern Woodlands	Horticulturalists	Yes	Direct	Acoustic	Individual	Turkeys	Lure* Luring
Seminole	North America	Eastern Woodlands	Horticulturalists	Yes	Direct	Visual	Individual	Deer	Deceive*
Shipibo	South America	Amazon and Orinoco	Horticulturalists	Yes	Direct	Acoustic	Individual	Birds	Imitat*
Sirionó	South America	Amazon and Orinoco	Hunter-gatherers	Yes	Direct	Acoustic	?	Tapir	Imitat*
Sirionó	South America	Amazon and Orinoco	Hunter-gatherers	Yes	Direct	Acoustic	?	Peccary	Imitat*
Sirionó	South America	Amazon and Orinoco	Hunter-gatherers	Yes	Direct	Acoustic	?	Monkey	Imitat*
Sirionó	South America	Amazon and Orinoco	Hunter-gatherers	Yes	Direct	Acoustic	?	Birds	Imitat*
Sirionó	South America	Amazon and Orinoco	Hunter-gatherers	Yes	Direct	Acoustic	Individual	Alligator	Imitat*
Sirionó	South America	Amazon and Orinoco	Hunter-gatherers	Yes	Indirect	Acoustic	Individual	Alligator	Imitat*
Slovenes	Europe	Southeastern Europe	Intensive Agriculturalists	Yes	Direct	Bait	Group	Pigs	Lure* Luring
Somali	Africa	Eastern Africa	Pastoralists	Yes	Direct	Acoustic	Individual	Birds	Imitat*
Somali	Africa	East Africa	Pastoralists	Yes	Indirect	Visual	Group	Antelope, Ostrich	Decoy*
Talamancans	Middle America and Caribbean	Central America	Other Subsistence Combinations	Yes	Direct	Acoustic	Individual	Mammals	Lure* Luring
Tanala	Africa	Southern Africa	Intensive Agriculturalists	Yes	Direct	Visual	Individual	Crayfish	Lure* Luring
Tehuelche	South America	Southern South America	Hunter-gatherers	Yes	Indirect	Bait	?	Guanaco	Decoy*
Ticuna	South America	Amazon and Orinoco	Primarily Hunter-gatherers	Yes	Indirect	Acoustic	Individual	Fish	Imitat*
Tikopia	Oceania	Polynesia	Horticulturalists	Yes	Direct	Fishing	Individual	Fish	Lure* Luring
Tinputz	Oceania	Melanesia	Other Subsistence Combinations	Yes	Direct	Fire Fishing	Group	Fish	Lure* Luring
Tinputz	Oceania	Melanesia	Other Subsistence Combinations	Yes	Indirect	Visual	Individual	Fish	Lure* Luring
Tiv	Africa	Western Africa	Horticulturalists	Yes	Direct	Acoustic	Individual	Birds	Imitat*

Tiv	Africa	Western Africa	Horticulturalists	Yes	Direct	Acoustic	Individual	Owls	Imitat*
Tiwi	Oceania	Australia	Hunter-gatherers	Yes	Indirect	Bait	Group	Kangaroos	Lure* Luring
Tiwi	Oceania	Australia	Hunter-gatherers	Yes	Direct	Visual	Group	Geese	Imitat*
Tlingit	North America	Northwest Coast and California	Hunter-gatherers	Yes	Direct	Acoustic	Individual	Deer	Decoy*
Tlingit	North America	Northwest Coast and California	Hunter-gatherers	Yes	Direct	Acoustic	Individual	Seals	Lure* Luring
Tlingit	North America	Northwest Coast and California	Hunter-gatherers	Yes	Indirect	Olfactory	Individual	Birds	Lure* Luring
Tlingit	North America	Northwest Coast and California	Hunter-gatherers	Yes	Indirect	Visual	Group	Seals	Decoy*
Tlingit	North America	Northwest Coast and California	Hunter-gatherers	Yes	Indirect	Visual	Individual	Groundhog	Lure* Luring
Tlingit	North America	Northwest Coast and California	Hunter-gatherers	Yes	Indirect	Visual	Individual	Fish	Lure* Luring
Tlingit	North America	Northwest Coast and California	Hunter-gatherers	Yes	Indirect	Visual	Group	Deer	Lure* Luring
Tonga	Africa	Southern Africa	Horticulturalists	Yes	Direct	Acoustic	?	Duiker	Lure* Luring
Tonga	Africa	Southern Africa	Horticulturalists	Yes	Indirect	Acoustic	Individual	Birds	Lure* Luring
Tonga	Africa	Southern Africa	Horticulturalists	Yes	Direct	Visual	Individual	Termites	Lure* Luring
Tongans	Oceania	Polynesia	Horticulturalists	Yes	Fishing	Fishing	Individual	Fish	Decoy*
Tongans	Oceania	Polynesia	Horticulturalists	Yes	Indirect	Visual	Individual	Birds	Decoy*
Tongans	Oceania	Polynesia	Horticulturalists	Yes	Indirect	Visual	Individual	Fish	Lure* Luring
Trobriands	Oceania	Melanesia	Other Subsistence Combinations	Yes	Direct	Acoustic	Individual	Birds	Imitat*
Trobriands	Oceania	Melanesia	Horticulturalists	Yes	Indirect	Acoustic	Individual	Sharks	Imitat*
Tubatubbal	North America	Northwest Coast and California	Hunter-gatherers	Yes	Direct	Visual	Individual	Deer	Decoy*
Tukano	South America	Amazon and Orinoco	Other Subsistence Combinations	Ambiguous	N/A	N/A	N/A	N/A	Imitat*
Tupinamba	South America	Eastern South America	Primarily Hunter-gatherers	Contextual	N/A	N/A	N/A	N/A	Deceive*
Ulithi	Oceania	Micronesia	Other Subsistence Combinations	Yes	Indirect	Fishing	?	Fish	Lure* Luring
Ute	North America	Southwest and Basin	Hunter-gatherers	Yes	Direct	Acoustic	Individual	Rabbit	Imitat*
Ute	North America	Southwest and Basin	Hunter-gatherers	Yes	Direct	Acoustic	Individual	Deer	Imitat*
Ute	North America	Southwest and Basin	Hunter-gatherers	Yes	?	Visual	?	Deer	Decoy*
Warao	South America	Amazon and Orinoco	Hunter-gatherers	Yes	Direct	Acoustic	Individual	Guinea Pigs	Imitat*
Warao	South America	Amazon and Orinoco	Hunter-gatherers	Yes	Direct	Acoustic	Group	Jaguar	Lure* Luring
Warao	South America	Amazon and Orinoco	Hunter-gatherers	Yes	Indirect	Acoustic	Individual	Birds	Decoy*
Warao	South America	Amazon and Orinoco	Hunter-gatherers	Yes	Fishing	Fishing	Individual	Fish	Deceive*
Warao	South America	Amazon and Orinoco	Hunter-gatherers	Yes	Direct	Fishing	Group	Fish	Lure* Luring
Western Apache	North America	Southwest and Basin	Primarily Hunter-gatherers	Yes	Direct	Acoustic	Individual	Deer	Lure* Luring
Western Apache	North America	Southwest and Basin	Primarily Hunter-gatherers	Yes	Direct	Visual	Group	Antelope	Imitat*
Western Woods Cree	North America	Arctic and Subarctic	Hunter-gatherers	Yes	Indirect	Bait	Individual	Beavers	Lure* Luring
Western Woods Cree	North America	Arctic and Subarctic	Hunter-gatherers	Yes	Indirect	Mixed	Individual	Birds	Decoy*
Wogeo	Oceania	Melanesia	Other Subsistence Combinations	Yes	Indirect	Fishing	?	Fish	Lure* Luring
Woleai Region	Oceania	Micronesia	Other Subsistence Combinations	Yes	Direct	Acoustic	Individual	Fish	Lure* Luring
Woleai Region	Oceania	Micronesia	Other Subsistence Combinations	Yes	Indirect	Fishing	Individual	Fish	Lure* Luring
Woleai Region	Oceania	Micronesia	Other Subsistence Combinations	Yes	Indirect	Visual	Individual	Fish	Lure* Luring
Xokleng	South America	Eastern South America	Hunter-gatherers	Yes	Direct	Acoustic	Individual	Tapir	Deceive*
Xokleng	South America	Eastern South America	Hunter-gatherers	Yes	Direct	Acoustic	Individual	Monkey	Imitat*
Xokleng	South America	Eastern South America	Hunter-gatherers	Yes	Indirect	Acoustic	Individual	Birds	Decoy*
Yahgan	South America	Southern South America	Hunter-gatherers	Yes	Direct	Acoustic	Individual	Seals	Lure* Luring
Yahgan	South America	Southern South America	Hunter-gatherers	Yes	Direct	Acoustic	Individual	Birds	Lure* Luring
Yahgan	South America	Southern South America	Hunter-gatherers	Yes	Indirect	Acoustic	Individual	Birds	Decoy*
Yahgan	South America	Southern South America	Hunter-gatherers	Yes	Fishing	Fishing	Individual	Fish	Deceive*
Yahgan	South America	Southern South America	Hunter-gatherers	Yes	Indirect	Visual	Individual	Birds	Deceive*
Yakut	Asia	North Asia	Other Subsistence Combinations	Yes	Indirect	Bait	Group	Reindeer	Decoy*
Yakut	Asia	North Asia	Other Subsistence Combinations	Yes	Indirect	Visual	Group	Bears	Decoy*
Yakut	Asia	North Asia	Other Subsistence Combinations	Yes	Indirect	Visual	Individual	Birds	Lure* Luring
Yanoama	South America	Amazon and Orinoco	Horticulturalists	Yes	Direct	Acoustic	Individual	Animals	Imitat*
Yapese	Oceania	Micronesia	Other Subsistence Combinations	Yes	Indirect	Bait	?	Crabs	Lure* Luring
Yapese	Oceania	Micronesia	Other Subsistence Combinations	Yes	Direct	Fire Fishing	Group	Fish	Lure* Luring
Yapese	Oceania	Micronesia	Other Subsistence Combinations	Yes	Indirect	Fishing	?	Fish	Lure* Luring
Yapese	Oceania	Micronesia	Other Subsistence Combinations	Yes	Indirect	Visual	Individual	Fish	Imitat*
Yokuts	North America	Northwest Coast and California	Hunter-gatherers	Yes	Direct	Bait	Group	Bears	Lure* Luring
Yokuts	North America	Northwest Coast and California	Hunter-gatherers	Yes	Indirect	Bait	Individual	Birds	Lure* Luring
Yokuts	North America	Northwest Coast and California	Hunter-gatherers	Yes	Indirect	Bait	Individual	Bears	Lure* Luring
Yokuts	North America	Northwest Coast and California	Hunter-gatherers	Yes	Indirect	Mixed	Individual	Eagles	Decoy*
Yokuts	North America	Northwest Coast and California	Hunter-gatherers	Yes	Indirect	Mixed	Individual	Birds	Decoy*
Yokuts	North America	Northwest Coast and California	Hunter-gatherers	Yes	Direct	Visual	Individual	Deer	Decoy*

Yokuts	North America	Northwest Coast and California	Hunter-gatherers	Yes	Direct	Visual	Group	Antelope	Decoy*
Yokuts	North America	Northwest Coast and California	Hunter-gatherers	Yes	Indirect	Visual	Individual	Waterfowl	Decoy*
Yuki	North America	Northwest Coast and California	Hunter-gatherers	Yes	Direct	Visual	Individual	Deer	Decoy*
Yurok	North America	Northwest Coast and California	Hunter-gatherers	Yes	Direct	Visual	Individual	Predators	Deceive*
Yurok	North America	Northwest Coast and California	Hunter-gatherers	Yes	Direct	Visual	?	Deer	Decoy*
Zulu	Africa	Southern Africa	Agro-Pastoralists	Yes	Direct	Acoustic	Individual	Birds	Imitat*
Zuni	North America	Southwest and Basin	Intensive Agriculturalists	Yes	Direct	Visual	Group	Deer	Imitat*

Data from 147 anthropological cultures in the Human Relations Area Files (eHRAF). Continent, Region, and Subsistence Type are properties of each society provided by eHRAF. Mimicry refers to presence/absence of aggressive mimicry. Type of Lure refers to signals coming directly from the hunter (direct) or from other sources, as with tools or other animals (indirect). Exploitation refers to the sensory system exploited by the hunter, Lure Context to whether hunters made the signal other animals (indirect). Exploitation refers to the sensory system exploited by the hunter, Lure Context to whether hunters made the signal as an individual or in groups, and Type of Meat refers to the animal deceived. Keywords are the terms used to prompt for eHRAF's search.
